# A tutorial on Bayesian model-averaged meta-analysis in JASP

**DOI:** 10.3758/s13428-023-02093-6

**Published:** 2023-04-26

**Authors:** Sophie W. Berkhout, Julia M. Haaf, Quentin F. Gronau, Daniel W. Heck, Eric-Jan Wagenmakers

**Affiliations:** 1 University of Amsterdam, Amsterdam, Netherlands; 2 Philipps University of Marburg, Marburg, Germany

**Keywords:** Bayesian model-averaging, Meta-analysis, Bayes factor, Posterior probability, Evidence synthesis

## Abstract

Researchers conduct meta-analyses in order to synthesize information across different studies. Compared to standard meta-analytic methods, Bayesian model-averaged meta-analysis offers several practical advantages including the ability to quantify evidence in favor of the absence of an effect, the ability to monitor evidence as individual studies accumulate indefinitely, and the ability to draw inferences based on multiple models simultaneously. This tutorial introduces the concepts and logic underlying Bayesian model-averaged meta-analysis and illustrates its application using the open-source software JASP. As a running example, we perform a Bayesian meta-analysis on language development in children. We show how to conduct a Bayesian model-averaged meta-analysis and how to interpret the results.

The standard method for aggregating empirical results across several studies is meta-analysis. Typically, the statistical analysis is conducted in the classical or frequentist framework (e.g.,Viechtbauer, 2010). However, Bayesian meta-analysis offers several advantages and has recently gained increasing interest in psychological science (e.g., van Erp, Verhagen, Grasman, & Wagenmakers, 2017; Nieuwenstein et al., 2015; Rouder, Haaf, Davis-Stober, & Hilgard, 2019). One Bayesian approach that seems particularly suited for meta-analysis is Bayesian model averaging (e.g., Gronau et al., 2017; Haaf, Hoogeveen, Berkhout, Gronau, & Wagenmakers, 2020; Scheibehenne, Gronau, Jamil, & Wagenmakers, 2017; Vohs et al., 2021). Here we present a tutorial on how to conduct Bayesian model-averaged meta-analysis with the user-friendly statistical software package JASP (JASP Team, 2020).

## Fixed effects versus random effects

Two key questions in every meta-analysis are whether there is evidence for an overall effect across studies and whether there is heterogeneity among study effects. To address these two questions, different statistical models for aggregating effect sizes across studies have been proposed. In the meta-analytic literature, these different approaches are consistently referred to as the fixed-effects^1^ model and the random-effects model. Let *δ*_*i*_ be the true effect size of the *i* th study and *μ* be the overall true effect size in the population. In the fixed-effects model, a single true effect size is estimated, which is assumed to be identical for all studies. This model can be expressed as *δ*_*i*_ = *μ*. In contrast, in the random-effects model, every study is assumed to have its own unique true effect size, albeit one that is likely to be similar in magnitude to that from the other studies. So, *δ*_*i*_ follows some distribution *g*(), which in most cases is a normal distribution with mean *μ* and standard deviation *τ* (i.e., the amount of heterogeneity), $\delta _{i} \sim \text {Normal}(\mu , \tau ^{2})$. Note that the fixed-effects model is obtained as a special case of the random-effects model by assuming that the true effect sizes have no variance (*τ* = 0).

**Fig. 1:**
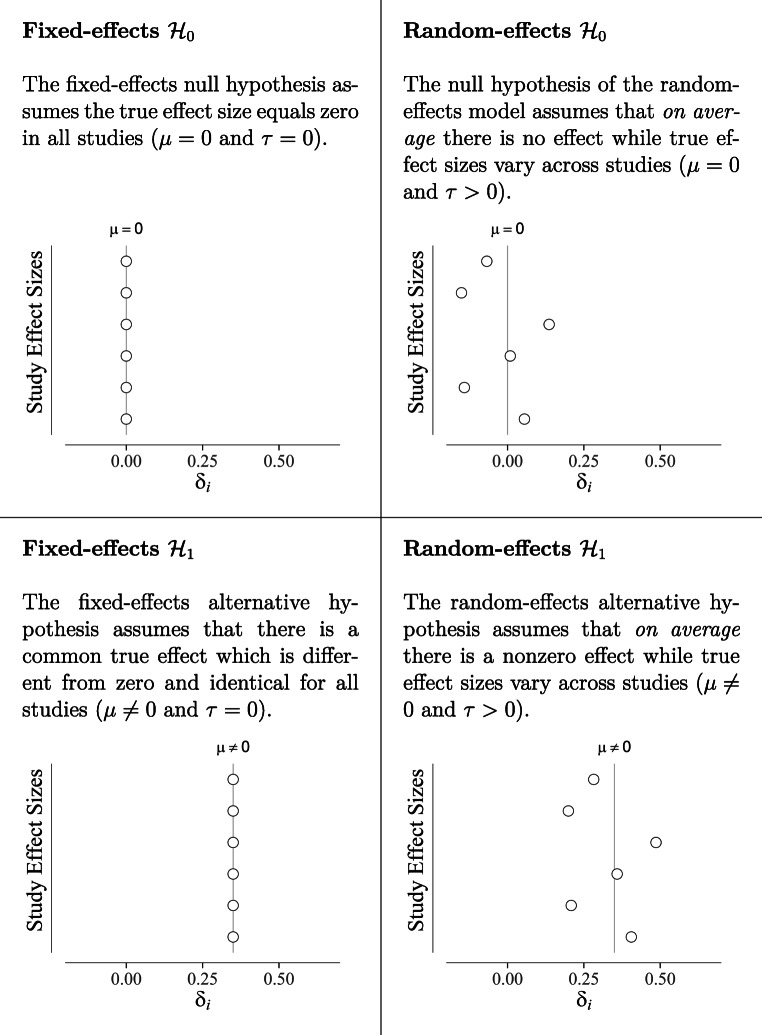
Explanation of the four meta-analytic models. The figures show the true effect sizes for six studies (*y*-axis) in accordance with the models. The *x*-axis depicts the study effect size *δ*_*i*_

When considering both fixed-effects and random-effects models, we can distinguish the four hypotheses shown in Fig. 1. Typically, the analyst first assesses the heterogeneity of study effects using heterogeneity statistics and then commits either to the fixed-effects model or to the random-effects model (Huedo-Medina, Sánchez-Meca, Marín-Martínez, & Botella, 2006)^2^. After making this commitment, the analyst usually proceeds with testing the null hypothesis that the overall effect is zero, $\mathcal H_{0}\!\!:\!\! \mu \!\! = 0$. The issue with this two-step approach, however, is that there may be considerable uncertainty in the initial decision on whether to rely on the fixed-effects models or on the random-effects models.

In practice, random-effects meta-analysis recently has been preferred (Serghiou & Goodman, 2019) and recommended (Hunter & Schmidt, 2000) over fixed-effects meta-analysis. The reason for this preference is the default assumption that studies entered in a meta-analysis must be different, and therefore, heterogeneity of effect sizes must be accounted for. While fixed-effects meta-analysis indeed leads to bias and too narrow confidence intervals in the presence of heterogeneity (Hunter & Schmidt, 2000), there are situations where fixed-effects meta-analysis might be the better choice (Borenstein, Hedges, Higgins, & Rothstein, 2010). First, if the studies included in the meta-analysis are direct replications or stem from the same lab, the fixed-effects assumptions might be more appropriate (Olsson-Collentine, Wicherts, & van Assen, 2020). Second, if the number of studies included in the meta-analysis is small, then study heterogeneity *τ* might not be precisely estimated leading to biases (Hedges & Pigott, 2004). For a more complete discussion of the advantages and disadvantages of the two approaches, please see (Borenstein et al., 2010).

We argue that the issue is not with the assumptions made by any of the two models, but with the *a priori* decision to consider only one of the models, whether or not heterogeneity statistics are used for making this choice. If the researcher chooses the fixed-effects model for null-hypothesis testing even though the random-effects model is also somewhat plausible, then the evidence for an effect is usually overestimated and true study heterogeneity is underestimated (i.e., it is assumed to be zero; Stanley & Doucouliagos, 2015). Likewise, if the researcher chooses the random-effects model for null-hypothesis testing even though the fixed-effects model is also somewhat plausible, then the evidence for an effect is often underestimated and true study heterogeneity is overestimated (Stanley & Doucouliagos, 2015). To address this issue, the analyst needs to take model uncertainty into account. This can be achieved by Bayesian model averaging (Hinne, Gronau, van den Bergh, & Wagenmakers, 2020; Hoeting, Madigan, Raftery, & Volinsky, 1999; Heck & Bockting, 2021; Kaplan & Lee, 2016).

## Bayesian model averaging

A Bayesian model-averaged meta-analysis considers the evidence for all four relevant models illustrated in Fig. 1: The fixed-effects null hypothesis, the fixed-effects alternative hypothesis, the random-effects null hypothesis, and the random-effects alternative hypothesis (for a recent extension see Maier, Bartoš, & Wagenmakers, 2022). By considering the uncertainty regarding these four statistical models simultaneously, it is possible to obtain the overall evidence for the null vs. the alternative hypothesis and the overall evidence for the existence vs. absence of heterogeneity. Moreover, the approach provides overall estimates for the parameters *μ* and *τ* by aggregating across the four models weighted by their plausibility. In Bayesian meta-analysis, model averaging has been successfully applied in several applications (Gronau et al., 2017; Haaf et al., 2020). For statistical details we refer the reader to Gronau et al., (2021).

In Bayesian statistics, the strength of evidence for statistical models is quantified by how well the different models can predict the observed data (Jeffreys, 1961; Kass and Raftery, 1995; Myung & Pitt, 1997). The predictive performance of a model is the marginal likelihood, that is, the prediction for the observed data averaged over the prior distribution of the parameters (Rouder & Morey, 2019). The marginal likelihood can be denoted as *p*(data|model). The ratio of one marginal likelihood over another is called the Bayes factor (BF; Etz & Wagenmakers, 2017, Jeffreys, 1961). For instance, a Bayes factor of the random-effects model over the fixed-effects model is

1 $$ \text{BF}_{rf} = \frac{p(\text{data} \mid \mathcal{H}_{random})}{p(\text{data} \mid \mathcal{H}_{fixed})}. $$

If the Bayes factor is, for example, equal to 6 this value indicates that the data are six times more likely under the random-effects model than under the fixed-effects model. Conversely, a BF_*rf*_ equal to 1/6, or approximately 0.17, indicates that the data support the fixed-effects model, as the data are six times less likely under the random-effects model than under the fixed-effects model. The Bayes factor informs us of the direction of evidence (i.e., which model made the better predictions) and the strength of that evidence (i.e., how much better the predictions are).

Bayes factors are particularly suitable for addressing the two key meta-analytic questions, as they are able to quantify evidence for and against the presence of an overall effect as well as heterogeneity. Although Bayes factors typically consider two models, Bayesian model-averaging allows the inclusion of multiple models with the so-called inclusion Bayes factor (Gronau et al., 2021). To understand the inclusion Bayes factor, we rearrange the terms of Bayes’ theorem to focus on an additional interpretation of the Bayes factor. Instead of taking the ratio of marginal likelihoods as in Eq. 1, we look at the relative change in beliefs about the models before observing the data to after observing data. The Bayes factor can then be expressed as

2 $$ \text{BF}_{rf} = \frac{p(\mathcal{H}_{random}) \mid \text{data})}{p(\mathcal{H}_{fixed}) \mid \text{data})} \Big/ \frac{p(\mathcal{H}_{random})}{p(\mathcal{H}_{fixed})}, $$

where $p({\mathscr{H}}_{random})$ and $p({\mathscr{H}}_{fixed})$ denote the prior model probabilities for the random- and fixed-effects model, i.e., a probability that represents one’s prior belief about the uncertainty of a model before seeing any data (Jeffreys, 1961; Kass & Raftery, 1995; Myung & Pitt, 1997), and $p({\mathscr{H}}_{random}) | \text {data})$ and $p({\mathscr{H}}_{fixed}) | \text {data})$ denote the posterior model probabilities of the random- and fixed-effects model, i.e., a probability that represents one’s belief about the uncertainty of a model after seeing data. The change in belief is quantified by dividing the posterior odds $\frac {p({\mathscr{H}}_{random}) | \text {data})}{p({\mathscr{H}}_{fixed}) | \text {data})}$ by the prior odds $\frac {p({\mathscr{H}}_{random})}{p({\mathscr{H}}_{fixed})}$, and we can also use this interpretation for the model-averaged inclusion Bayes factor.

The inclusion Bayes factor for effect size compares all models that assume an effect to be present (i.e., fixed-effects ${\mathscr{H}}_{1}$ and random-effects ${\mathscr{H}}_{1}$) to all models that assume an effect to be absent (i.e., fixed-effects ${\mathscr{H}}_{0}$ and random-effects ${\mathscr{H}}_{0}$), that is

3 $$ \text{BF}_{10} = \frac{p(\mathcal{H}_{1, fixed}) \mid \text{data}) + p(\mathcal{H}_{1, random}) \mid \text{data})}{p(\mathcal{H}_{0, fixed}) \mid \text{data}) + p(\mathcal{H}_{0, random}) \mid \text{data})} \Big/ \frac{p(\mathcal{H}_{1, fixed}) + p(\mathcal{H}_{1, random})}{p(\mathcal{H}_{0, fixed}) + p(\mathcal{H}_{0, random})}. $$

Similarly, the inclusion Bayes factor for heterogeneity compares all models that assume effect sizes vary across studies (i.e., random-effects ${\mathscr{H}}_{0}$ and random-effects ${\mathscr{H}}_{1}$) to all models that assume study effect sizes are identical (i.e., fixed-effects ${\mathscr{H}}_{0}$ and fixed-effects ${\mathscr{H}}_{1}$), that is

4 $$ \text{BF}_{rf} = \frac{p(\mathcal{H}_{0, random}) \mid \text{data}) + p(\mathcal{H}_{1, random}) \mid \text{data})}{p(\mathcal{H}_{0, fixed}) \mid \text{data}) + p(\mathcal{H}_{1, fixed}) \mid \text{data})} \Big/ \frac{p(\mathcal{H}_{0, random}) + p(\mathcal{H}_{1, random})}{p(\mathcal{H}_{0, fixed}) + p(\mathcal{H}_{1, fixed})}. $$

Hence, one can examine the overall evidence for the presence or absence of an effect and of heterogeneity, without first having to select a subset of models.

Besides the evidence for the presence or absence of an overall effect *μ*, researchers may be interested in its size (i.e., how large is the treatment effect, assuming it exists?). In Bayesian statistics, parameter estimates like *μ* come from a posterior distribution, which according to Bayes’ theorem can be expressed as

5 $$ p(\mu \mid \text{data}) = \frac{p(\text{data} \mid \mu) p(\mu)}{p(\text{data})}, $$

where *p*(data|*μ*) is the likelihood of the data given parameter *μ*, *p*(data) is the marginal likelihood of the data under the model, and *p*(*μ*) is the prior distribution for *μ*, i.e., a probability distribution that represents one’s prior belief about the size and uncertainty of *μ* before seeing any evidence (Jeffreys, 1961; Kass & Raftery, 1995; Myung & Pitt, 1997). The uncertainty of the parameter estimates are typically represented as a credible interval, which represents the posterior probability that the parameter estimate lies within said interval. For example, a 95% credible interval means that there is a posterior probability of 95% that the true parameters falls within this interval. Note the difference to a classical frequentist 95% confidence interval, which means that if one were to calculate confidence intervals for an infinite number of repeated samples, 95% of these intervals would include the true parameter.

Model-averaging is useful here as well. Specifically, the fixed-effects ${\mathscr{H}}_{1}$ provides an estimate for *μ* (*τ* is assumed zero) and the random-effects ${\mathscr{H}}_{1}$ provides estimates for both *μ* and *τ*. When there is uncertainty concerning these two models, Bayesian model-averaging yields a single estimate of *μ* that is a weighted average of the estimates from the two rival models, with the largest weight assigned to the model that predicted the data best.^3^

**Fig. 2:**
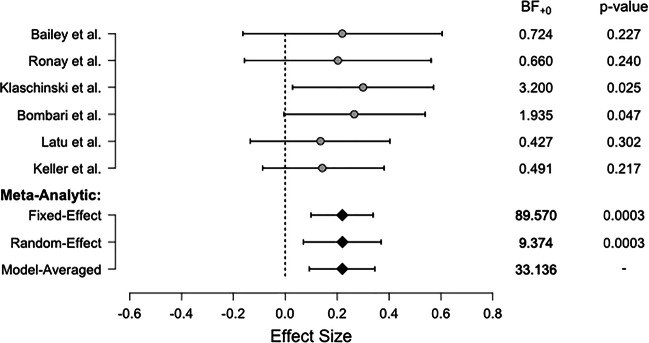
Forest plot for the Bayesian model-averaged meta-analysis by Gronau et al., (2017). Shown are the estimated effect sizes per study (*points*) and the estimates of the overall effect sizes per model (fixed-effect, random-effects, and model-averaged; diamonds). The corresponding Bayes factors and *p* values are given on the right. Figure available at http://tinyurl.com/kz2jpwb under CC license https://creativecommons.org/licenses/by/2.0/

To illustrate the advantages of the Bayesian model averaging approach, consider the meta-analysis conducted by Gronau et al., (2017). This meta-analysis concerned the effect of “power posing” on felt power. The analysis included six preregistered studies that measured self-reported felt power after participants adopted a high-power body posture in the experimental condition and a low-power body pose in the control condition. The forest plot in Fig. 2 shows the medians of the posterior distributions together with the 95*%* highest density intervals for all effect size estimates.^4^ The right-hand side of the figure shows the Bayes factors in favor of the alternative directional hypothesis ${\mathscr{H}}_{+}$ and the *p* value.

It is clear from the results that the individual studies do not provide much evidence for the presence of an effect when considered separately. The individual Bayes factors are close to 1 and thus do not strongly support either the null or the alternative hypothesis, although two of the six studies have a significant *p* value. However, all of the effect-size estimates indicate a positive effect. The fixed-effects Bayes factor is BF_+ 0_ = 89.57 and thus indicates strong evidence in favor of a positive effect. The Bayes factor for the random-effects model also indicates evidence for a positive effect, but the strength of evidence is considerably lower, BF_+ 0_ = 9.37. Instead of drawing a conclusion based on the fixed-effects comparison only or based on the random-effects comparison only, model averaging takes the model uncertainty into account and yields in-between evidence, in this case, BF_+ 0_ = 33.14. The model-averaged effect size point estimate is *μ* = 0.22, 95*%* HDI [0.09, 0.34].

## Advantages and challenges

Compared to a standard classical meta-analysis, a Bayesian model-averaged meta-analysis offers several advantages. First of all, the Bayesian analysis allows evidence to be quantified for two or more hypotheses. Hence, it is possible to determine the degree to which the data support a certain hypothesis over another. Specifically, one may obtain evidence in favor of the null hypothesis (when it outpredicts the alternative hypothesis), evidence in favor of the alternative hypothesis (when it outpredicts the null hypothesis), and absence of evidence (when both hypotheses predict the data about equally well; Keysers, Gazzola, & Wagenmakers, 2020). In contrast, the *p* value from classical methods cannot discriminate evidence of absence from absence of evidence.

Second, a Bayesian model-averaged meta-analysis naturally accommodates the uncertainty across multiple candidate models. This is particularly likely to be advantageous when the number of studies is small, such that no single model is dominant. In these cases, the Bayesian method prevents the overconfidence that comes from eliminating the model-selection step (Hinne et al., 2020), both with respect to models themselves and with respect to their parameters. The classical meta-analysis, however, does not provide a straightforward method to produce model-averaged parameter estimates (O’Hagan & Forster, 2004 p. 174; but see Burnham & Anderson, 2002).

Third, a Bayesian model-averaged meta-analysis allows researchers to monitor the evidence as studies accumulate indefinitely (Rouder, 2014; Scheibehenne, Jamil, & Wagenmakers, 2016; Wagenmakers, Gronau, & Vandekerckhove, 2018). Usually, the sampling plan in meta-analyses is not under the control of an experimenter, and this means that classical methods are vulnerable to the problem of multiple comparisons (but see Schnuerch & Erdfelder, 2020).

Finally, a Bayesian model-averaged meta-analysis enables researchers to take into account prior knowledge. This prior knowledge may reflect expectations concerning effect size (Vohs et al., 2021) or heterogeneity (van Erp et al., 2017). In addition, the theory under scrutiny often implies a certain direction of the effect – for instance, the theory may stipulate it to be positive. In Bayesian inference, this substantive knowledge can be accommodated by adjusting the prior distribution. Including more detailed knowledge in the prior distribution allows for a more diagnostic test. Classical statistics cannot accommodate detailed prior knowledge.

Bayesian model-averaging for meta-analysis is a relatively new methodology, and consequently, it has yet to be included in popular statistics programs. The method has recently been implemented in the R package metaBMA (Heck, Gronau, & Wagenmakers, 2019). However, many students and researchers in the social sciences rely on statistical software with a point-and-click graphical user interface. In the present paper, we showcase a recent implementation of Bayesian model-averaged meta-analysis in JASP, which relies on the metaBMA R package. The software JASP is an open-source statistics program with an intuitive graphical user interface (e.g., JASP Team, 2020; Love et al., 2019; Ly, van den Bergh, Bartoš, & Wagenmakers, 2021; Wagenmakers et al., 2018). We explain how Bayesian model-averaged meta-analysis can be conducted, interpreted, and reported.

## Example: pointing and language development

In a classical meta-analysis comprised of 12 studies with a total of 319 children, Colonnesi et al., (2010) examined the concurrent relation between pointing (indicating something with one’s finger) and language development. The results of each study are summarized in Table 1.

**Table 1:** Results for the 12 studies included in the Colonnesi et al., (2010) meta-analysis on the concurrent relation between pointing and language development

	*r*	SE	N
Murphy (1978)	0.310	0.186	32
Bates et al. (1979)	0.250	0.213	25
Dobrich & Scarborough (1984)	0.400	0.229	22
Harris et al. (1995)	0.700	0.567	6
Mundy & Gomes (1998)	0.320	0.218	24
Morales et al. (2000)	0.500	0.229	22
Rowe (2000)	0.640	0.154	45
Franco & Gagliano (2001)	0.720	0.186	32
Fasolo & d’Odorico (2002)	0.490	0.156	44
Rodrigo et al. (2006)	0.510	0.444	8
Locke (2007)	0.820	0.406	9
Rowe & Goldin-Meadow (2008)	0.610	0.146	50

*Note.* Data retrieved from http://metalab.stanford.edu/

The Colonnesi et al., (2010) meta-analysis showed a large overall effect size that was also statistically significant (*r* = .52,*z* = 8.80,*p* < .001). Moreover, a test of homogeneity indicated that the hypothesis of homogeneity could not be rejected at the *α* = .05 level (i.e., *Q*(9) = 15.53,*p* = .077). Based on these results, Colonnesi et al., (2010) concluded that there was a strong concurrent relationship between the pointing gesture and language development for infants. In the next section, we show how to conduct and interpret a Bayesian model-averaged meta-analysis of the data from Colonnesi et al., (2010) in JASP.

## Implementation in JASP

Before proceeding with the analysis, we first need to load the data file in JASP. The .jasp file containing the data used in this example together with the analysis input and output are available at https://osf.io/84gbu/. The data file must contain a column with effect sizes and another column with the corresponding standard errors, with each row corresponding to a specific study. Alternatively, the standard error column can be replaced with two columns for the lower and upper bound of a 95% confidence interval of the effect size. In this case, standard errors are computed internally assuming a normal distribution as sampling distribution. An optional column with study labels can be used to customize tables and figures.

JASP assumes that the effect size measure is scaled in such a way that zero corresponds to the null effect and the measure can take on any value on the real line. This is the case for many common effect size measures in social science such as Cohen’s *d*, Hedges’ *g*, Fisher’s *z*, and the log odds ratio (LOR). Other effect-size measures should be transformed prior to the analysis. For example, on the odds-ratio scale, a value of 1 corresponds to the null effect (i.e., no change in the odds). In this case, a log transformation of each study’s odds ratio and the corresponding standard error can be used to obtain effect sizes on the right scale (i.e., LOR). Similarly, in correlational research, it is common to use the Pearson correlation coefficient *r* as a measure of effect size. While *r* = 0 indeed corresponds to the null hypothesis, correlation coefficients are restricted to the interval from − 1 to 1. Our meta-analytic models assume that effect sizes can range from minus to plus infinity (i.e., they have full support). Therefore, it is necessary to transform each study’s correlation coefficient and the associated standard error to Fisher’s *z*, the standardized correlation coefficient. In our example, the reported effect sizes and corresponding standard errors are in terms of the Pearson correlation coefficient *r*. Appendix A shows how JASP can be used to transform *r* to *z*.

**Fig. 3:**
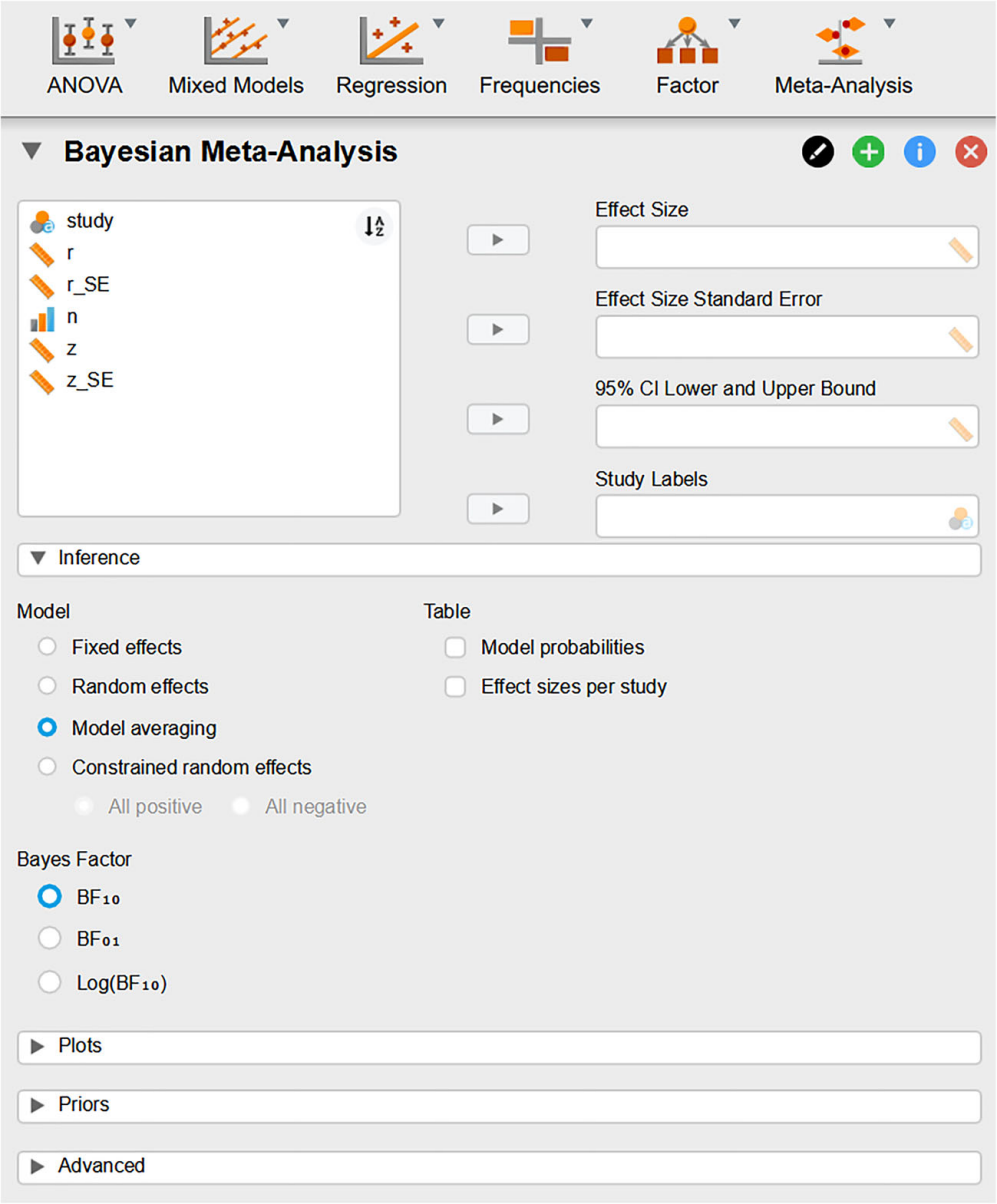
JASP screenshot of the input panel for the Bayesian meta-analysis module. The variables from the data file are listed in the *top-left box* and can be moved to the appropriate boxes on the *top-right*. Below the data input boxes are various options for inference and displaying results. More detailed options are available under the drop-down subsections Prior, Plots, and Advanced

Having transformed the data to the right scale, we proceed to activate the ‘Meta-Analysis’ module. To do so, we navigate to the top right of the JASP application and click on the large blue ‘+’ sign (not shown) and select the ‘Meta-Analysis’ module. A meta-analysis button is added to the ribbon; selecting it shows the option ‘Bayesian Meta-Analysis’. Selecting the ‘Bayesian Meta-Analysis’ option produces the graphical user interface shown in Fig. 3.

### Prior settings

**Fig. 4:**
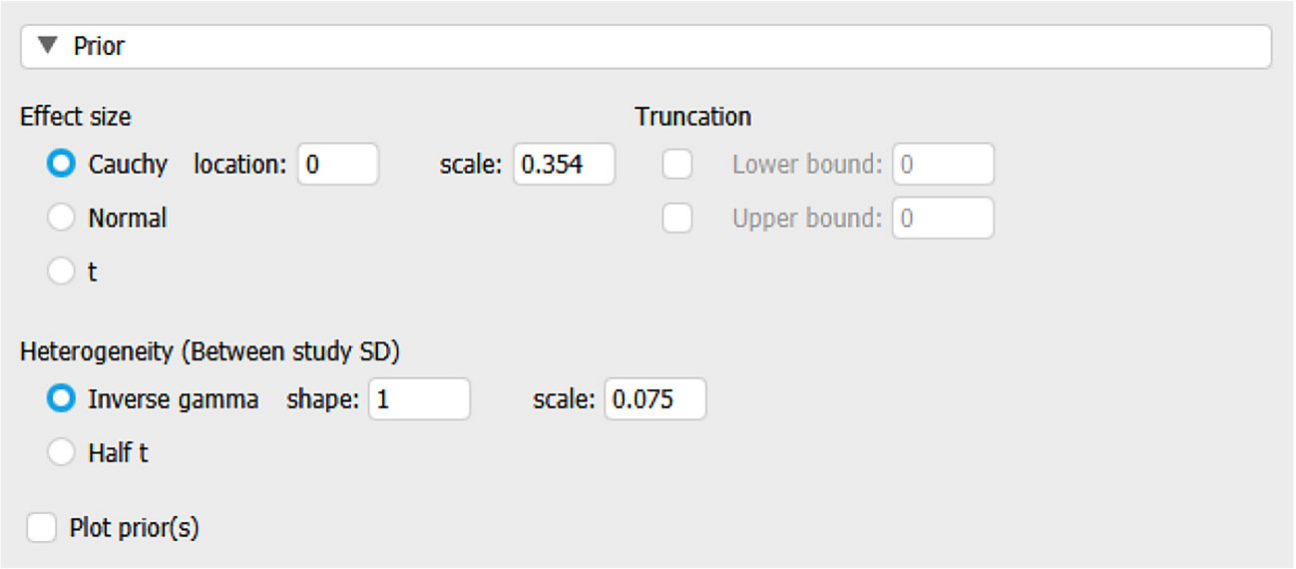
JASP screenshot of the prior distribution options for effect size and heterogeneity in the Bayesian meta-analysis module

The typical workflow in JASP is to drag the relevant variables into the appropriate boxes using the mouse cursor – which yields immediate output – and only then adjust the default settings of an analysis. However, here we examine and adjust the prior settings first. Figure 4 shows the input fields that allow analysts to specify the relative plausibility of different values for effect size and heterogeneity before having seen the data – in other words, prior distributions.

For the overall effect size *μ*, the default prior distribution in JASP is a Cauchy distribution with location 0 and scale 0.707: $\mu \sim \text {Cauchy}(0, 0.707)$ (cf. Morey and Rouder, 2018). When choosing a prior, it is important to take into account the scaling of the effect size (Haaf & Rouder, 2021). In particular, the Cauchy prior with scale 0.707 is commonly applied to the Cohen’s *d* scale (Gronau et al., 2021); however, we have used a Fisher’s *z* transformation, and Fisher’s *z* values are about twice as small as Cohen’s *d* values. Hence, we specify a zero-centered Cauchy distribution on *μ* with a scale of 0.707/2 = 0.354: $\mu \sim \text {Cauchy}(0, 0.354)$.

**Fig. 5:**
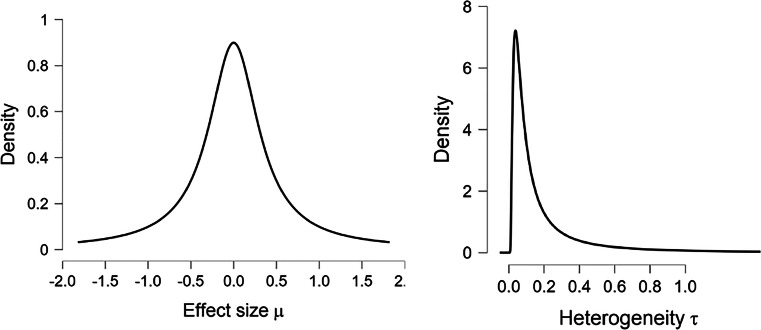
Prior distributions for the Bayesian meta-analysis of the studies identified by Colonnesi et al., (2010) on the concurrent relation between pointing and language development. *Left panel*: $\mu \sim \text {Cauchy}(0, 0.354)$. *Right panel*: $\tau \sim \text {Inv-Gamma}(1, 0.075)$. Note that these priors are on the Fisher’s *z* scale, with values about twice as small as Cohen’s *d* values. Figures from JASP

For heterogeneity *τ*, the default prior is an inverse gamma with shape 1 and scale 0.15, $\tau \sim \text {Inv-Gamma}(1, 0.15)$. This prior was proposed by Gronau et al., (2017) based on an empirical review of effect-size heterogeneity by van Erp et al., (2017). As before, this prior distribution needs to be adjusted to take into account that our values are on the Fisher’s *z* scale. Consequently, we assign *τ* an inverse gamma distribution with shape 1 and scale 0.075: $\tau \sim \text {Inv-Gamma}(1, 0.075)$. These priors can be plotted to assess whether the distributions represent the analyst’s beliefs, that is, whether most prior mass is placed on reasonable values. The prior distributions specified above are shown in Fig. 5.

### The default results

With the priors specified on the correct scale, we can proceed to conduct the analysis. We place the effect size variable ‘z’ in the Effect Size box, the standard error variable ‘z_SE’ in the Effect Size Standard Error box, and the study label variable ‘study’ in the Study Labels box. Doing so immediately starts an analysis. As shown in Fig. 3, by default this analysis relies on model averaging. Researchers may change this setting and instead opt for a fixed-effects or a random-effects meta-analysis only. Moreover, the random-effects model can be constrained to a nested model in which all of the true effect sizes across studies (i.e., the random effects) are either positive or negative, thus resembling a stronger version of the more common one-sided hypothesis that the *average* effect size is positive or negative (Rouder et al., 2019).^5^ Researchers may retain the default setting of Bayesian model averaging when they are unwilling to fully commit, from the outset, to either the fixed-effects model or the random-effects model. Below we continue to discuss the results obtained from model-averaging.

#### Posterior estimates per model

Table 2 presents the main results: the posterior distribution for *μ* and *τ* per model and the corresponding Bayes factors. The columns ‘Mean’, ‘SD’, ‘Lower 95% Credible Interval’ and ‘Upper 95% Credible Interval’ summarize the posterior distribution for either *μ* or *τ*. For instance, the first row shows that the posterior mean for *μ* in the fixed-effects model is 0.578 (on Fisher’s *z* scale), with a standard deviation of 0.061 and a 95% credible interval that ranges from 0.461 to 0.698. The fixed-effects model assumes *τ* = 0, so no posterior distribution is shown. The BF_10_ = 5.625e + 19 value indicates that the data are 5.625 ⋅ 10^19^ times more likely under the fixed-effects ${\mathscr{H}}_{1}$ than under the fixed-effects ${\mathscr{H}}_{0}$ – overwhelming evidence for the presence of an effect if a fixed-effects model is assumed.

**Table 2:** Posterior estimates per model for the Bayesian meta-analysis of the studies identified by (Colonnesi et al., 2010) on the concurrent relation between pointing and language development

		95% Credible Interval				
		Mean	SD	Lower	Upper	BF_10_
Fixed effects	*μ*	0.578	0.061	0.461	0.698	5.625e + 19
Random effects	*μ*	0.572	0.071	0.427	0.708	38503.011
	*τ*	0.096	0.067	0.019	0.270	0.854^*a*^
Averaged	*μ*^*b*^	0.575	0.067	0.438	0.700	83574.393
	*τ*^*c*^					0.854

Table from JASP

*Note.* *μ* and *τ* are the group-level effect size and standard deviation, respectively

^a^ Bayes factor of the random effects ${\mathscr{H}}_{1}$ over the fixed effects ${\mathscr{H}}_{1}$

^b^ Posterior estimates are based on the models that assume an effect to be present. The Bayes factor is based on all four models: fixed- and random-effects ${\mathscr{H}}_{1}$ over the fixed- and random-effects ${\mathscr{H}}_{0}$.

^c^ Model-averaged posterior estimates for *τ* are not yet available, but will be added in the future

For the random-effects model, the posterior distribution for *μ* is similar to that of the fixed-effects model. In addition, the random-effects model also features a posterior distribution for *τ*. The BF_10_ value of 38,503.011 on the second row indicates that the data are about 38,500 times more likely under the random-effects ${\mathscr{H}}_{1}$ than under the random-effects ${\mathscr{H}}_{0}$. This is still overwhelming evidence for the presence of an effect, but considerably less so than that obtained under a fixed-effects assumption. On the third row, the BF_10_ value of 0.854 indicates that the data are about 1/0.854 ≈ 1.17 times more likely under the fixed-effects ${\mathscr{H}}_{1}$ than under the random-effects ${\mathscr{H}}_{1}$. In other words, under the assumption that the effect is present, the data provide almost no evidence for the assertion that the effect is either fixed or random. This is more informative than the results of the classical frequentist analysis, which indicated that the null hypothesis of homogeneity could not be rejected (*p* = .077).

The fourth row shows, first, a summary of the model-averaged posterior distribution for *μ*. The averaging here occurs over the fixed-effects ${\mathscr{H}}_{1}$ and the random-effects ${\mathscr{H}}_{1}$, that is, the models that assume *μ* to be present. This model-averaged distribution for *μ* falls in between the posterior under the fixed-effects ${\mathscr{H}}_{1}$ and the posterior under the random-effects ${\mathscr{H}}_{1}$. The fourth row also shows the model-averaged Bayes factor for the presence of an effect; with BF_10_ = 83,574.393 this Bayes factor falls in between that from the fixed-effects comparison and the random-effects comparison.

#### Model probabilities and effect sizes

Apart from the main output table, two additional tables are available upon demand. The first table shows the prior and posterior model probabilities; Table 3 provides the results for the Bayesian meta-analysis of the studies identified by Colonnesi et al., (2010). The fixed-effects and random-effects null models both have a posterior model probability close to zero, indicating that a null effect is highly unlikely. Among the two remaining alternative models, the fixed-effects ${\mathscr{H}}_{1}$ has received just a little more support from the data than the random-effects alternative model, meaning that the posterior probability for the fixed-effects ${\mathscr{H}}_{1}$ edges out that for the random-effects ${\mathscr{H}}_{1}$ (i.e., 0.539 vs. 0.461). The fact that the two posterior probabilities are so similar indicates that, after having seen the data, there remains considerable uncertainty about the presence or absence of study heterogeneity, which may be due to the small number of studies. Because of this uncertainty it is prudent to quantify evidence for or against an overall effect by averaging across the fixed and random-effects models.

**Table 3:** Prior and posterior model probabilities for the Bayesian model-averaged meta-analysis of the studies identified by Colonnesi et al., (2010) on the concurrent relation between pointing and language development

	Prior	Posterior
Fixed ${\mathscr{H}}_{0}$	0.250	9.587e-21
Fixed ${\mathscr{H}}_{1}$	0.250	0.539
Random ${\mathscr{H}}_{0}$	0.250	1.197e-5
Random ${\mathscr{H}}_{1}$	0.250	0.461

Table from JASP

The second on-demand table shows the observed and estimated effect sizes per study; Table 4 provides the results for the Bayesian meta-analysis of the studies identified by Colonnesi et al., (2010). The estimated effect sizes are summarized by their posterior means and 95% credible intervals. Note that the estimated per-study effect sizes are closer to the average effect size (i.e., *μ* = 0.575) than the observed effect sizes. This shrinkage effect can also be appreciated visually through a forest plot, which is the topic of the next section.

**Table 4:** Observed and estimated effect sizes (i.e., Fisher’s *z*) per study for the Bayesian model-averaged meta-analysis of the studies identified by Colonnesi et al., (2010) on the concurrent relation between pointing and language development

	Estimated			
	Observed	Mean^a^	Lower^a^	Upper^a^
Murphy (1978)	0.321	0.521	0.255	0.712
Bates et al. (1979)	0.255	0.517	0.213	0.720
Dobrich & Scarborough (1984)	0.424	0.551	0.300	0.753
Harris et al. (1995)	0.867	0.583	0.328	0.849
Mundy & Gomes (1998)	0.332	0.532	0.258	0.734
Morales et al. (2000)	0.549	0.570	0.339	0.782
Rowe (2000)	0.758	0.622	0.440	0.848
Franco & Gagliano (2001)	0.908	0.645	0.456	0.936
Fasolo & d’Odorico (2002)	0.536	0.565	0.368	0.742
Rodrigo et al. (2006)	0.563	0.574	0.306	0.828
Locke (2007)	1.157	0.612	0.379	0.919
Rowe & Goldin-Meadow (2008)	0.709	0.613	0.441	0.818

Table from JASP

^a^ Posterior mean and 95% credible interval estimates from the random-effects model

#### Forest plot

A standard forest plot shows the observed effect sizes plus confidence intervals per study ordered in rows. On the left, study labels indicate the study. In the middle, points with error bars illustrate the effect size and confidence interval of each study. On the right, the exact values of the effect size and confidence interval are displayed. Underneath the study-specific information are the results from the meta-analysis.

**Fig. 6:**
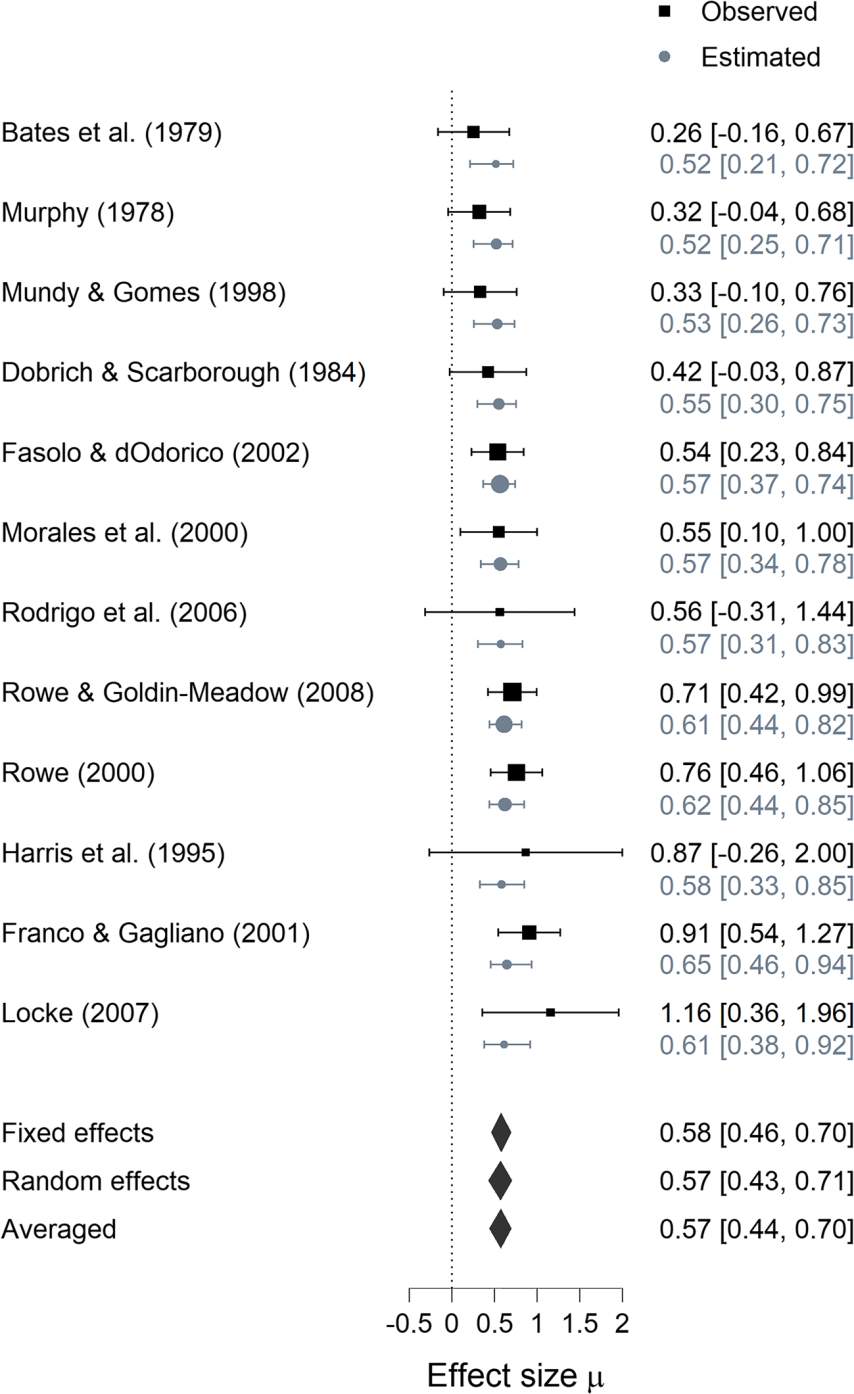
Forest plot for the studies identified by Colonnesi et al., (2010) on the concurrent relation between pointing and language development. Observed per-study effect sizes (i.e., Fisher’s *z*) with 95% confidence intervals are shown in *black*; estimated per-study effect sizes with 95% credible intervals are shown in *gray*. Figure from JASP

In addition to the standard forest plot, which show the observed effect sizes per study, JASP also offers the option to display the estimated effect sizes per study. Figure 6 shows a forest plot with observed per-study effect sizes in black and estimated per-study effect sizes in gray. The estimated effect sizes are shrunk towards the group mean (cf. Table 4). The meta-analytic estimates for the fixed, random, and averaged models are shown at the bottom of the figure. The averaged estimate is a weighted mean of the fixed and random-effects estimates where the weighting is determined by the posterior probability of the two ${\mathscr{H}}_{1}$ models.

#### Cumulative forest plot

**Fig. 7:**
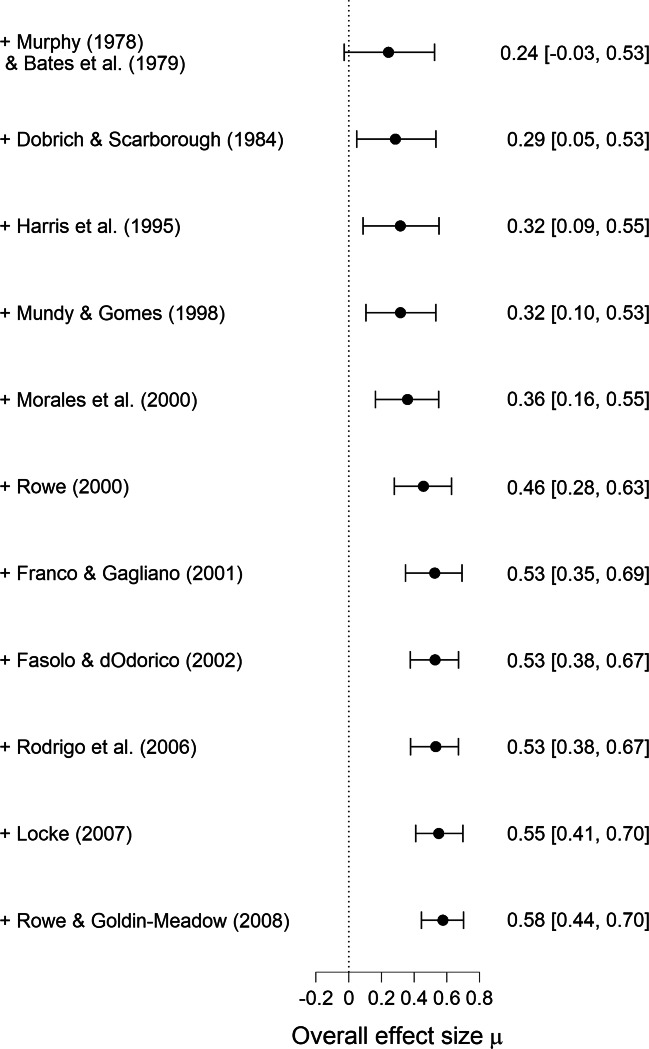
Cumulative forest plot for the studies identified by Colonnesi et al., (2010) on the concurrent relation between pointing and language development. Each consecutive row shows the model-averaged estimate of *μ* (with a 95% credible interval) after adding the associated study to the analysis. The *bottom row* shows the result for the complete data set. Figure from JASP

The cumulative forest plot shows how the posterior model-averaged estimates are updated as studies are added to the analysis one-by-one. Figure 7 shows the cumulative forest plot for the Colonnesi et al., (2010) example. The studies are placed in chronological order, such that the posterior distributions reflect the increase in knowledge as the studies come in over time. In JASP, the row order of the data provides the order in which the studies are added to the cumulative forest plot. The top row shows the posterior estimate based on two studies, as this is the minimum number for a random-effects meta-analysis (and therefore also for model-averaging). The bottom row shows the posterior estimate based on the complete data set, with all studies included. As the studies accumulate, the posterior distribution for the mean effect size *μ* narrows, indicating an increase in confidence regarding the plausible values for *μ*.

#### Prior and posterior plots

**Fig. 8:**
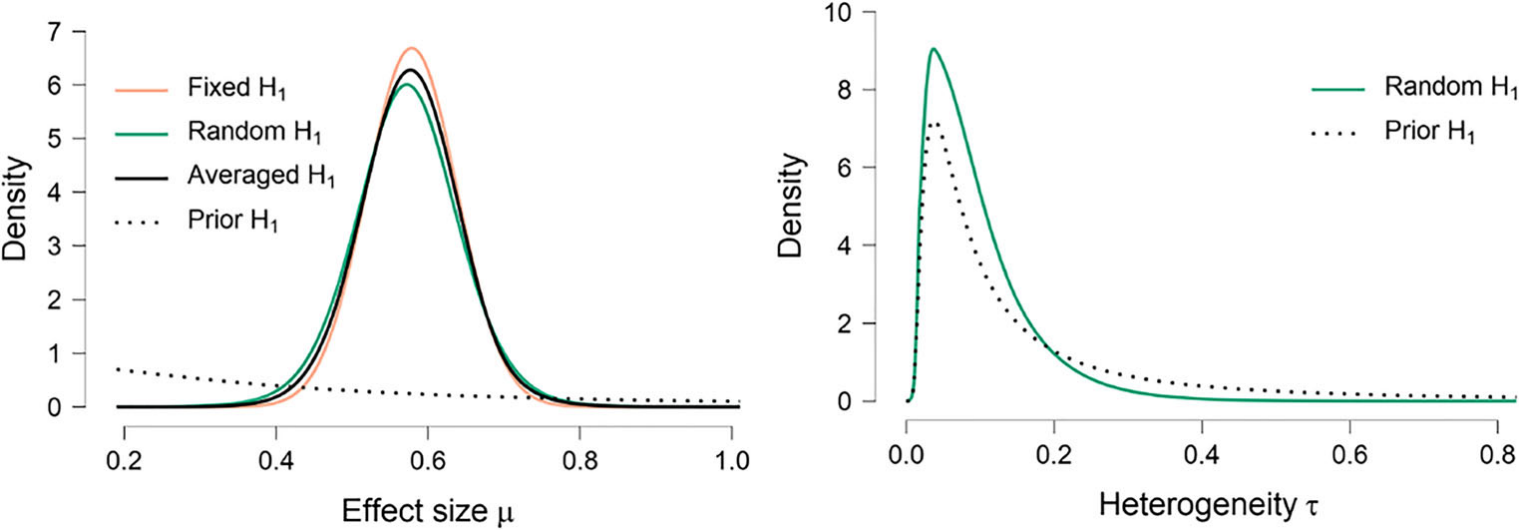
Posterior distributions (*solid lines*) and prior distributions (*dotted lines*) for the Bayesian meta-analysis of the studies identified by Colonnesi et al.,(2010) on the concurrent relation between pointing and language development. *Left panel*: prior and posterior distribution on effect size (for the fixed effects, random effects, and model-averaged alternative models). *Right panel*: prior and posterior distribution on heterogeneity (for the random effects alternative model). Figures from JASP

Two other plots shows the prior and posterior distribution for the overall effect size and the study heterogeneity parameters; Fig. 8 provides the results for the Bayesian meta-analysis of the studies identified by Colonnesi et al., (2010).

These plots can be used to gauge how much the data have changed the relative plausibility of the different parameter values. In addition, the posterior distributions provide a more complete picture of the uncertainty than the numerical summary using the posterior mean and 95% credible interval. The left panel shows, at a glance, that the Fisher’s *z* effect size is highly likely to lie in the range from 0.4 to 0.8; note that expressed in terms of Cohen’s *δ*, the effect would be about twice as small. The right panel shows that the posterior distribution for heterogeneity *τ* is somewhat more narrow than the prior distribution, but has not changed markedly. This reflects our finding above that the Bayes factor for the fixed- vs. the random-effects model is close to one, meaning that we cannot draw any conclusions about the variance of effect sizes. The right-skew of the posterior distribution arises because the heterogeneity parameter is bounded from below by zero; this is a prominent feature of the distribution that is difficult to appreciate from a numerical summary alone.

#### Sequential analyses

We consider two sequential analyses, in which the analysis outcome is updated one study at a time. The order in which the studies are added is given by the rows in the data set; the most natural organization is chronologically. These sequential analyses are conceptually similar to the cumulative forest plot, except that the analysis outcome is not an effect-size estimate, but rather the evidence (i.e., the Bayes factor) or belief (i.e., posterior probabilities) for the different models.

**Fig. 9:**
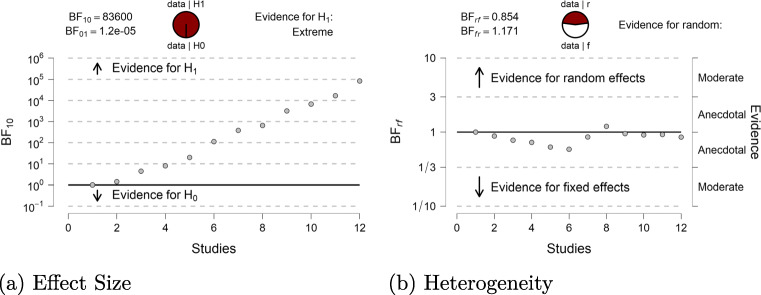
The flow of evidence: model-averaged meta-analytic Bayes factor sequential analyses for effect size (*left panel*) and heterogeneity (*right panel*). Data are based on the studies identified by Colonnesi et al., (2010) on the concurrent relation between pointing and language development. The panels show how evidence accumulates when studies are added one-by-one. Panels from JASP

First, Fig. 9 shows the flow of evidence, that is, the development of the Bayes factors as the studies accumulate.^6^ The left panel shows the model-averaged Bayes factor for the presence vs. absence of an effect. Every study increases the strength of evidence in favor of there being an effect; after six studies, the Bayes factor already exceeds 100. The right panel shows the model-averaged Bayes factor for heterogeneity vs. homogeneity. The Bayes factor remains close to one throughout the study series, meaning that the data are almost completely uninformative with respect to the presence of heterogeneity.

**Fig. 10:**
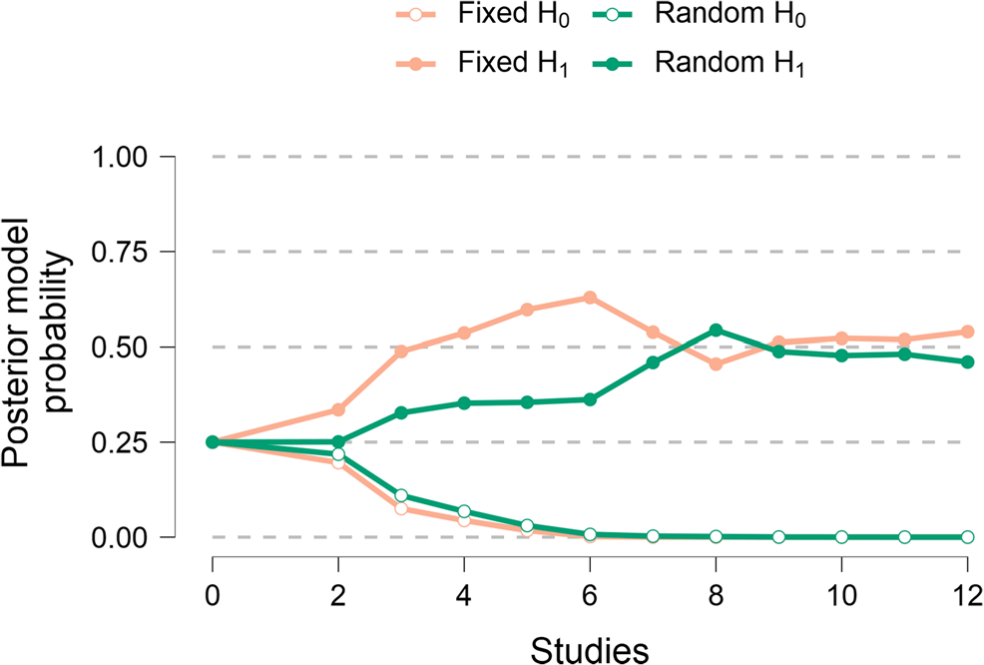
The flow of belief: sequential analysis of posterior model probabilities for the four meta-analytic models. Data are based on the studies identified by Colonnesi et al., (2010) on the concurrent relation between pointing and language development. The *lines* show how the posterior model probabilities fluctuate when studies are added one-by-one. Figure from JASP

Second, Fig. 10 shows the flow of belief, that is, the development of posterior model probabilities as studies accumulate. For the first few studies, the fixed-effects alternative hypothesis is preferred. After seven studies, the fixed-effects alternative hypothesis and the random-effects alternative hypothesis are about equally plausible, and this situation remains unchanged as the remaining studies are added one-by-one. After including six studies, both the fixed-effects null-hypothesis and the random-effects null-hypothesis have posterior model probabilities near zero and are effectively out of contention.

### Advanced settings

**Fig. 11:**
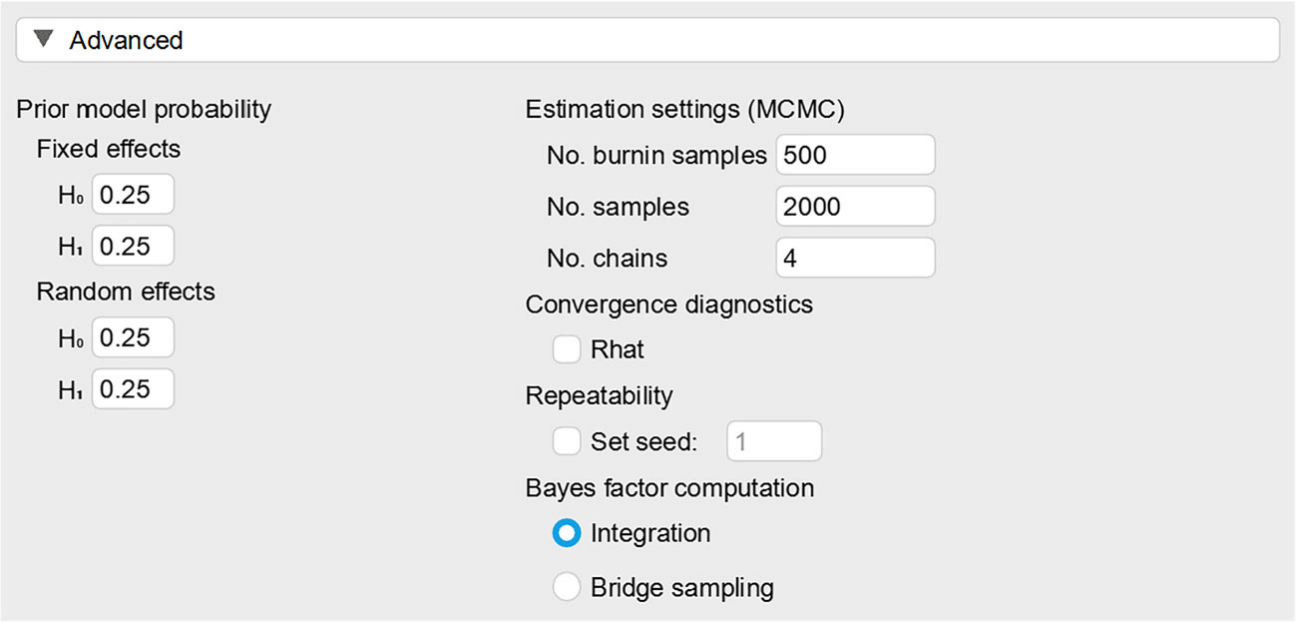
Advanced settings for the Bayesian meta-analysis. See text for details

Figure 11 shows the GUI for the advanced settings. The prior model probabilities concern the fixed-effects ${\mathscr{H}}_{0}$, the fixed-effects ${\mathscr{H}}_{1}$, the random-effects ${\mathscr{H}}_{0}$, and the random-effects ${\mathscr{H}}_{1}$. If, before seeing the data, there is good reason to believe that any of these models are less likely than others, their prior probabilities may be changed instead of using the default value of 0.25. For example, when one believes that the random-effects models are twice as plausible as the fixed-effects models, the prior model probabilities of the fixed-effects ${\mathscr{H}}_{0}$ and ${\mathscr{H}}_{1}$ can be set to 0.167, and the prior model probabilities of the random-effects ${\mathscr{H}}_{0}$ and ${\mathscr{H}}_{1}$ can be set to 0.333. The four prior probabilities should add to 1. If this is not the case, JASP will rescale the values to enforce this restriction. Note that changing the prior model probabilities may affect the value of the inclusion Bayes factor (for details see Gronau et al., 2021, Appendix).

Under estimation settings, there are options to change the behavior of the Markov chain Monte Carlo (MCMC) routine. MCMC is a sampling method that allows one to estimate a posterior distribution by drawing a large number of randomly generated values from this distribution. We need MCMC procedures in Bayesian estimation because posterior distributions are often difficult if not impossible to derive analytically. The samples in a Markov chain are generated sequentially, where each sample depends on the previous sample but not on the samples before that. Because of this dependence, the initial samples need to be ignored, often called ‘burn-in’ or ‘warm-up’, as the starting point of the chain may not be representative of the posterior distribution (for a gentle introduction to MCMC, see van Ravenzwaaij, Cassey, & Brown, 2018). When estimation problems occur, it may help to increase the number of chains, burn-in samples, and iterations. Furthermore, JASP offers an option to show the convergence diagnostic R-hat for the parameter estimates of the fixed- and random-effects model, and the individual-study effect-size estimates. R-hat is the potential scale reduction statistic, where a value close to 1 indicates that the MCMC chains have converged, more specifically, a value smaller than 1.05 can be seen as an indicator of convergence, although a more conservative cut-off of 1.01 has been proposed (Vehtari, Gelman, Simpson, Carpenter, & Bürkner, 2021).

Under Bayes factor computation one may select either Integration or Bridge sampling (e.g., Gronau et al., 2017; Gronau, Singmann, & Wagenmakers, 2020). The bridge sampling method is slower and less precise than the numerical integration method; however, the integration method is not robust under extreme priors or data (e.g., very high prior precision, very small standard error; see also Heck et al.,, 2019). When one chooses bridge sampling for computing the Bayes factor, one may rerun the analysis a few times to gauge the robustness of the estimated posterior probabilities. For the analysis of the Colonnesi et al., (2010) data we used the default advanced settings.

### Prior robustness analysis

The JASP implementation above shows that the Bayesian model-averaged meta-analysis requires two prior settings: the prior model probabilities of the four models (fixed-effects ${\mathscr{H}}_{0}$, fixed-effects ${\mathscr{H}}_{1}$, random-effects ${\mathscr{H}}_{0}$, and random-effects ${\mathscr{H}}_{1}$) and the prior parameter distributions for the overall effect size *μ* and the study heterogeneity *τ*. When there is uncertainty about these prior settings, we recommend conducting a prior robustness analysis, that is, trying out other reasonable prior settings and see how this impacts the results. In this section, we illustrate how one can perform a prior robustness analysis using the example above.

#### Prior model probabilities

So far, we used equal prior model probabilities, that is, a prior probability of 0.25 for each of the four models. This resulted in posterior model probabilities of almost zero for the null models and about equal probabilities for the alternative models, see Table 3. To see how sensitive the results of the Bayesian model-averaged meta-analysis are to the prior model probabilities, we can conduct the same analysis with different reasonable prior model probabilities. Thus, we have to adjust the prior model probabilities in the advanced settings in JASP. For example, we could see what happens if the null models are twice as likely *a priori* as the alternative models, or if the random-effects models are twice as likely *a priori* as the fixed-effects models, or vice versa.

Table 5 shows the posterior model probabilities for different settings of prior model probabilities. We see that the posterior probabilities are similar when assuming equal prior probabilities and when assuming prior probabilities that favor the null models. However, when the prior model probabilities favor either the fixed- or the random-effects model, the posterior probabilities show the same pattern for the alternative models (while the posterior probabilities for the null models remain close to zero). This effect of the prior probabilities on the posterior probabilities is expected here since the data provide no evidence for or against heterogeneity. Thus, for all prior model probabilities settings, our conclusion would be that the alternative models are more likely than the null models. However, which alternative model (fixed-effects ${\mathscr{H}}_{1}$ or random-effects ${\mathscr{H}}_{1}$) has a higher posterior probability depends on the prior settings.

**Table 5:** Posterior model probabilities for four different prior model probabilities settings

		$p({\mathscr{H}})$ Favors			
Probability	Hypothesis	None	Null	Random	Fixed
$p({\mathscr{H}})$	Fixed ${\mathscr{H}}_{0}$	**0.250**	0.333	0.167	0.333
	Fixed ${\mathscr{H}}_{1}$	**0.250**	0.167	0.167	0.333
	Random ${\mathscr{H}}_{0}$	**0.250**	0.333	0.333	0.167
	Random ${\mathscr{H}}_{1}$	**0.250**	0.167	0.333	0.167
$p({\mathscr{H}} \mid \text {data})$	Fixed ${\mathscr{H}}_{0}$	**9.587e-21**	1.917e-20	6.563e-21	1.246e-20
	Fixed ${\mathscr{H}}_{1}$	**0.539**	0.539	0.369	0.701
	Random ${\mathscr{H}}_{0}$	**1.197e-5**	2.393e-5	1.638e-5	7.773e-6
	Random ${\mathscr{H}}_{1}$	**0.461**	0.461	0.631	0.299

*Note.* The results of our main analysis are highlighted in bold

Moreover, Table 6 shows that the overall effect size estimate *μ* is similar for all four prior settings. The only relevant difference concerns the inclusion Bayes factor, which does not change when assuming either prior probabilities favoring none or the null model. However, compared to the default model probabilities, the inclusion Bayes factor is about 1.4 times smaller for the prior probabilities favoring the random-effects model and about 1.5 times larger for the prior probabilities favoring the fixed-effects model. This means that the fixed-effects model has obtained more evidence for an effect compared to the random-effects model. However, for all these prior model probabilities settings, our conclusion regarding *μ* would be the same: There is considerable evidence for an effect. Note that in cases where the amount of evidence is generally smaller, a difference by a factor of 1.5 could result in a different interpretation of the inclusion Bayes factor.

**Table 6:** Model-averaged effect size estimates for four prior model probabilities settings

				95% Credible Interval		
$p({\mathscr{H}})$ Favors		Mean	SD	Lower	Upper	BF_10_
None	*μ*	**0.575**	**0.067**	**0.438**	**0.700**	**83574.393**
Null	*μ*	0.575	0.066	0.442	0.702	83574.393
Random	*μ*	0.574	0.068	0.437	0.705	61038.702
Fixed	*μ*	0.577	0.063	0.452	0.700	128645.776

*Note.* *μ* is the group-level effect size. Posterior estimates are based on the models that assume an effect to be present. The Bayes factor is based on all four models: fixed- and random-effects ${\mathscr{H}}_{1}$ over the fixed- and random-effects ${\mathscr{H}}_{0}$. The results of our main analysis are highlighted in bold

#### Prior distribution *μ*

In the example, we used the prior distribution of the overall effect size $\mu \sim \text {Cauchy}(0, 0.354)$, see Fig. 4. To see whether the results of the meta-analysis are robust against the choice of this prior distribution, we can try out different reasonable distribution settings. For instance, one may assume a less informed, wider prior distribution such as $\mu \sim \text {Cauchy}(0, 0.707)$, or a more informed, narrower prior distribution such as $\mu \sim \text {Cauchy}(0, 0.177)$. Table 7 shows the posterior model probabilities for the three different prior distributions of *μ* which are all very similar.

**Table 7:** Posterior model probabilities for three different prior distributions of *μ*

		$p({\mathscr{H}} \mid \text {data})$		
Hypothesis	$p({\mathscr{H}})$	Cauchy(0, 0.177)	Cauchy(0, 0.354)	Cauchy(0, 0.707)
Fixed ${\mathscr{H}}_{0}$	0.25	1.512e-20	**9.587e-21**	8.765e-21
Fixed ${\mathscr{H}}_{1}$	0.25	0.537	**0.539**	0.542
Random ${\mathscr{H}}_{0}$	0.25	1.887e-5	**1.197e-5**	1.094e-5
Random ${\mathscr{H}}_{1}$	0.25	0.463	**0.461**	0.458

*Note.* The results of our main analysis are highlighted in bold

We expect the prior distribution for *μ* to have the most impact on the effect-size estimates. Table 8 shows that, as expected, the wider the prior distribution, the larger the estimate for *μ*, and the larger the Bayes factor. However, these differences are relatively small and do not change the substantive conclusions.

**Table 8:** Model-averaged effect size estimates for three different prior distributions of *μ*

				95% Credible Interval		
Prior		Mean	SD	Lower	Upper	BF_10_
Cauchy(0, 0.177)	*μ*	0.571	0.066	0.440	0.698	53002.651
Cauchy(0, 0.354)	*μ*	**0.575**	**0.067**	**0.438**	**0.700**	**83574.393**
Cauchy(0, 0.707)	*μ*	0.580	0.065	0.451	0.706	91413.823

*Note.* *μ* is the group-level effect size. Posterior estimates are based on the models that assume an effect to be present. The Bayes factor is based on all four models: fixed- and random-effects ${\mathscr{H}}_{1}$ over the fixed- and random-effects ${\mathscr{H}}_{0}$. The results of our main analysis are highlighted in bold

#### Prior distribution *τ*

We can also try other reasonable prior distributions for the heterogeneity that are more and less informed. In the example, we used the prior distribution of the heterogeneity $\tau \sim \text {Inv-Gamma}(1, 0.075)$, so a wider, less informed distribution would be $\tau \sim \text {Inv-Gamma}(1, 0.150)$, and a narrower, more informed distribution is $\tau \sim \text {Inv-Gamma}(1, 0.038)$. Table 9 shows the posterior model probabilities for these three prior distributions of *τ*. When the prior distribution becomes wider, the fixed-effects alternative model has a higher and the random-effects model a lower posterior probability. This is expected as a wider prior means that there is less prior probability for heterogeneity (compared to narrower priors), which is part of the random-effects model. Furthermore, Table 10 shows the estimates and Bayes factors for *τ*. Even though the estimate for heterogeneity *τ* is largest for the widest prior (*τ* = 0.130), the 95% credible interval shows more uncertainty. The Bayes factor for the widest prior indicates that the data are about 1/0.665 ≈ 1.53 times more likely under the fixed-effects ${\mathscr{H}}_{1}$ than under the random-effects ${\mathscr{H}}_{1}$, which can be interpreted as anecdotal evidence for the fixed-effects model. Thus, with this wider prior for heterogeneity, the results slightly favor the fixed-effects over the random-effects alternative model, which indicates that the results are sensitive to the prior settings. However, the difference in evidence is still too small to change our conclusion.

**Table 9:** Posterior model probabilities for three different prior distributions of *τ*

		$p({\mathscr{H}} \mid \text {data})$		
Hypothesis	$p({\mathscr{H}})$	Inv-Gamma(1, 0.038)	Inv-Gamma(1, 0.075)	Inv-Gamma(1, 0.150)
Fixed ${\mathscr{H}}_{0}$	0.25	9.109e-21	**9.587e-21**	1.074e-20
Fixed ${\mathscr{H}}_{1}$	0.25	0.512	**0.539**	0.604
Random ${\mathscr{H}}_{0}$	0.25	6.161e-6	**1.197e-5**	2.341e-5
Random ${\mathscr{H}}_{1}$	0.25	0.488	**0.461**	0.396

*Note.* The results of our main analysis are highlighted in bold

**Table 10:** Heterogeneity estimates for three different prior distributions of *τ*

				95% Credible Interval		
Prior		Mean	SD	Lower	Upper	BF_*rf*_
Inv-Gamma(1, 0.038)	*τ*	0.069	0.059	0.010	0.227	0.952
Inv-Gamma(1, 0.075)	*τ*	**0.096**	**0.067**	**0.019**	**0.270**	**0.854**
Inv-Gamma(1, 0.150)	*τ*	0.130	0.070	0.035	0.302	0.655

*Note.* *τ* is the group-level standard deviation. The inclusion Bayes factor is the random effects ${\mathscr{H}}_{0}$ and ${\mathscr{H}}_{1}$ over the fixed effects ${\mathscr{H}}_{0}$ and ${\mathscr{H}}_{1}$. The results of our main analysis are highlighted in bold

### Example report

In this section, we provide an example report of our Bayesian meta-analysis for the data by Colonnesi et al., (2010). We follow van Doorn and colleagues’ suggestions for transparent reporting of Bayesian analyses (van Doorn et al., 2021).

To investigate the relationship between pointing and language development, we conducted a Bayesian model-averaged meta-analysis using data from Colonnesi et al., (2010). This analysis features four models or hypotheses: (1) the fixed-effects null-hypothesis; (2) the fixed-effects alternative hypothesis; (3) the random-effects null-hypothesis; and (4) the random-effects alternative hypothesis. We analyzed the data with JASP (JASP Team, 2020). An annotated .jasp file, including plots, tables, data, and input options, is available at https://osf.io/84gbu/.

#### Descriptive summary

Table 1 summarizes the data from each of the 12 studies, and Fig. 6 shows the associated forest plot. All studies have a positive Fisher’s *z* score, with values ranging from 0.255 to 1.157.

#### Testing for heterogeneity

Firstly, we examined the inclusion Bayes factor for heterogeneity. This Bayes factor pits the two random-effects hypotheses against the two fixed-effects hypotheses. All four hypotheses were given a prior probability of 0.25 (Table 3), reflecting a position of prior impartiality. The Bayes factor indicated that there is little evidence for the presence or absence of study heterogeneity. Specifically, BF_*rf*_ = 0.85 (Table 2), which means that the data are approximately equally likely under the random-effects hypotheses and the fixed-effects hypotheses.

#### Testing for overall effect size

Secondly, we examined the inclusion Bayes factor for effect size. This Bayes factor pits the two alternative hypotheses against the two null hypotheses. As in the test for heterogeneity, all four hypotheses were given a prior probability of 0.25. The results are shown in Table 3. There is decisive evidence for the presence of an effect, BF_10_ = 83,574.39, which means that the data are over 83,000 times more likely under the effect-present hypotheses than under the effect-absent hypotheses.

#### Parameter estimation

Finally, we discuss the results of parameter estimation. For the estimation of heterogeneity, the across-study standard deviation *τ* was given an inverse gamma prior distribution, $\tau \sim \text {Inv-Gamma}(1, 0.075)$. Under the random-effects alternative hypothesis, the posterior mean of the heterogeneity parameter *τ* equals 0.096 with a 95% credible interval ranging from 0.019 to 0.270. The large width of the credible interval indicates that there remains considerable uncertainty about the degree of heterogeneity across studies.

For effect-size estimation, we assumed a Cauchy distribution with $\mu \sim \text {Cauchy}(0, 0.354)$ as a prior for the parameter *μ* (i.e., Fisher’s *z*). Because the data did not provide convincing evidence for preferring either the fixed-effects or the random-effects model, we average the posterior distribution of *μ* over both models.

Figure 8 shows the posterior distributions for all models. The model-averaged posterior falls in between the posterior distributions of the fixed-effects and the random-effects model. The fixed-effects posterior mean *μ* is equal to 0.578, 95*%* CI[0.461,0.698], and the random-effects posterior mean *μ* is equal to 0.572, 95*%* CI[0.427,0.708]. Note that the credible interval for the random-effects model is somewhat more uncertain then the fixed-effects model. The mean of the model-averaged posterior distribution for *μ* is equal to 0.575 with a 95% credible interval that ranges from 0.438 to 0.700 (see Table 2). Although there is some uncertainty about the exact size of the effect, it is almost certainly very large: in terms of Cohen’s *d*, the point estimate of effect size is 1.214 with a 95% central credible interval ranging from 0.904 to 1.517.

#### Prior sensitivity

To check whether these results are robust to alternative, reasonable prior settings, we conducted a prior robustness analysis with different prior model probabilities and prior distributions. With different prior model probabilities, the posterior model probabilities ranged from 0.369 to 0.701 for the fixed-effects alternative model and from 0.299 to 0.631 for the random-effects model, depending on which model was favored in the prior settings. Moreover, the posterior estimates for the effect size *μ* were similar, ranging from 0.575 to 0.577, with inclusion Bayes factors ranging from 61,039 to 128,646. With different prior distributions for *μ*, the posterior estimates for the effect size were similar, ranging from 0.571 to 0.580, with inclusion Bayes factors ranging from 53,002 to 91,413. Finally, with different prior distributions for *τ*, the posterior estimates for heterogeneity varied slightly, ranging from 0.069 to 0.130, with inclusion Bayes factors ranging from 1.050 to 1.527.

## Conclusions

In this tutorial, we demonstrated how to conduct a Bayesian meta-analysis in the open-source statistical software JASP. We explained the basic concepts underlying Bayesian model averaging for meta-analysis, the required data structure, and the available settings. Most importantly, we described how to interpret the results from a Bayesian model-averaged meta-analysis in order to draw valid substantive conclusions.

The present paper also highlights the advantages of Bayesian inference for meta-analysis compared to the classical frequentist approach. First, the Bayesian analysis allows researchers to quantify the strength of evidence for one model over another. In our example, the classical analysis did not allow us to distinguish between evidence for the absence of heterogeneity vs. the absence of evidence, whereas the Bayesian analysis showed that there was absence of evidence. By simultaneously taking into account both the random-effects model and the fixed-effects model, Bayesian model-averaging incorporates the inherent uncertainty associated with the model-selection process. Second, Bayesian analyses seamlessly extent to scenarios in which the studies come in over time, necessitating a study-by-study update of knowledge. This ability is manifested in the cumulative forest plot and in the sequential plots that show the flow of evidence and the flow of the posterior model probabilities.

Overall, this paper shows that JASP provides a convenient and efficient way to perform a Bayesian meta-analysis. The software facilitates a straightforward interpretation of the results even for researchers who are not (yet) experts on Bayesian inference and statistics.

## Figures and Tables

**Fig. 12:**
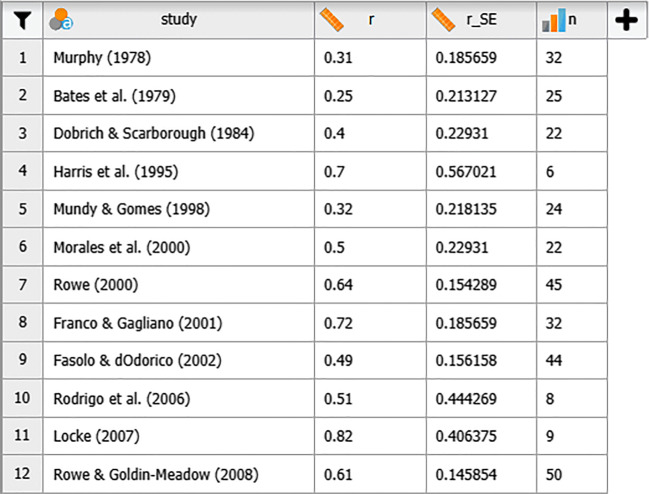
JASP spreadsheet data view for the Colonnesi et al., (2010) meta-analysis. Clicking the ‘+’ sign on the top right activates the ‘create computed column’ functionality

**Fig. 13:**
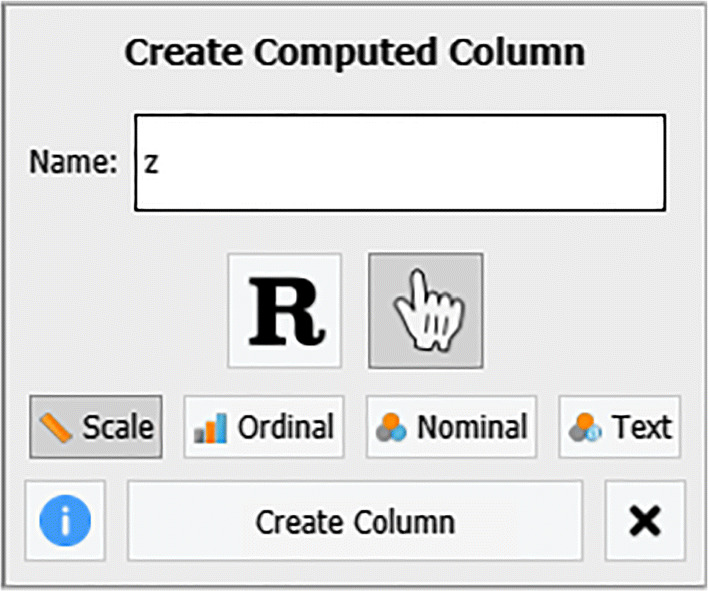
The JASP pop-up menu that initiates the creation of a computed column. Users are asked to name the new column and determine the method of computation (i.e., R or the drag-and-drop interface)

**Fig. 14:**
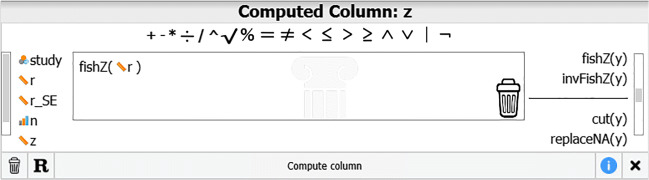
The JASP drag-and-drop interface to transform correlation coefficients *r* to the Fisher’s *z* scale

**Fig. 15:**
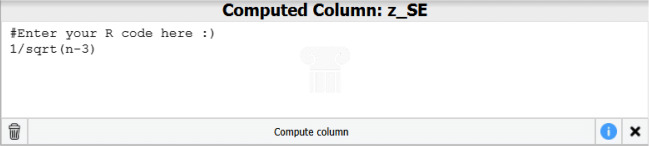
The JASP R interface to obtain standard errors on the Fisher’s *z* scale

**Fig. 16:**
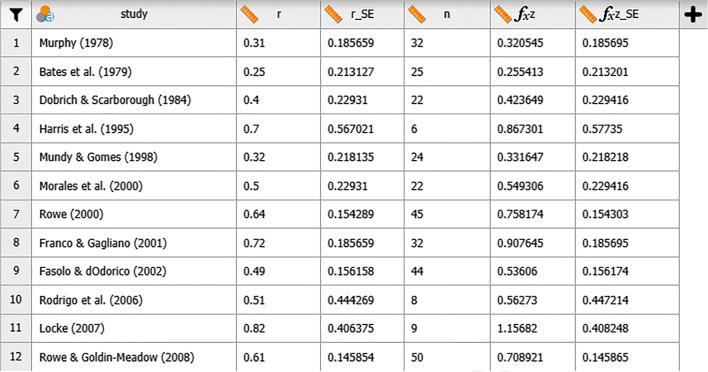
JASP spreadsheet data view for the Colonnesi et al., (2010) meta-analysis showing the Fisher’s *z* transformed variables

## Data Availability

The data set analyzed during the current study are available in the MetaLab repository, http://metalab.stanford.edu/.

## Appendix A: Transforming data in JASP

JASP allows the transformation of variables through the ‘create computed column’ functionality. There are two ways to compute a new column: either by using a drag-and-drop formula interface or by writing R code. In this section we explain how to use both methods to obtain the Fisher’s *z* transformed coefficients and standard errors.

The Fisher’s *z* transformation of the correlation coefficient *r* is given by

$$ z = \frac{1}{2} \ln \left( \frac{1+r}{1-r} \right). $$

The corresponding standard error of Fisher’s *z* depends only on study sample size *n*:

$$ \frac{1}{\sqrt{n-3}}.$$

In order to create a computed column, we can click on the ‘+’ symbol next to the final column on the top right in the data spreadsheet view (cf. Fig. 12). The resulting pop-up menu, shown in Fig. 13, asks us to name the new column and select the method of computation.

### Transforming data with the drag-and-drop interface

The JASP drag-and-drop interface is activated by selecting the hand-pointer icon from the JASP pop-up menu shown in Fig. 13. The drag-and-drop interface, shown in Fig. 14, presents the spreadsheet variables to the left, a range of frequently used functions to the right, and a series of relational operators on top.

In order to apply the Fisher’s *z* transform to the correlations, we first scroll down the function list and find the formula for the Fisher’s *z* transformation, denoted ‘fishZ(y)’. We drag and drop the formula to the input window and drop it. We then drag and drop the correlation variable *r* into the brackets of the function in the input window. Clicking ‘Compute column’ will then make the Fisher’s *z* transformed variable appear in the data spreadsheet view.

### Transforming data with the R interface

In order to apply the Bayesian meta-analysis, it is also necessary to obtain the standard error on the Fisher’s *z* scale. The relevant formula $1/\sqrt {n-3}$ can be put together using the operators shown on top of Fig. 14, but here we demonstrate how to obtain the Fisher’s *z* standard errors using R code. First we select the R symbol in the pop-up menu shown in Fig. 13. This activates an input window, shown in Fig. 15, that accepts R code. The column names can be used as variables in the R code.^7^ Entering the formula and clicking ‘Compute column’ makes the Fisher’s *z* transformed variable appear in the data spreadsheet view.

Figure 16 shows the data view with the newly computed columns. After the transformations are complete one can conduct the Bayesian model-averaged meta-analysis as explained in the main text.

## Appendix B: Constrained random-effects

In addition to Bayesian model-averaged meta-analysis we also implemented a second Bayesian method for meta-analysis in JASP using ordinal constraints, which also relies on the metaBMA package (Heck et al., 2019). This approach is based on Rouder et al., (2019) and Haaf and Rouder (2021), and we refer interested readers to these papers for a detailed introduction. The key idea is that psychological theories often distinguish between negative, zero, and positive effects. This distinction poses a problem for conventional meta-analysis. For example, suppose we want to meta-analyze a set of studies on the mere exposure effect where half the studies have a large positive effect corresponding to the notion that repeatedly encountering an item leads to increased liking of the item, and half the studies have a large negative effect corresponding to the notion that repeatedly encountering an item leads to decreased liking of the item. The meta-analytic average of this analysis might be close to zero. Yet, we cannot interpret the average in any meaningful way as it does not describe any of the included studies well.

Motivated by the concern that qualitatively different outcomes correspond to different psychological processes we may first want to answer the question whether all true study effects are qualitatively the same, that is, all effects are in the same direction, or that some effects are qualitatively different, that is, they are in opposite directions. If all true effects are plausibly in the same direction, then the average across these effects is much more interpretable as an overall effect of a common phenomenon. This issue is also discussed in the clinical literature as quantitative vs. qualitative interactions (Gail & Simon, 1985; Pan & Wolfe, 1997).

To answer the question whether all studies show an effect in the same direction we may conduct a constrained random-effects analysis in JASP (this analysis is also included in the .jasp file available at https://osf.io/84gbu/). The first step is to choose the option *constrained random effects* in the user interface of the Bayesian meta-analysis module (Fig. 3). We may then also indicate whether the expected direction of effects is positive or negative. Dependent on this choice JASP will perform a model comparison between a model where all effect sizes are constrained to be positive (or negative), and the fixed- and random-effects models described before. As part of this analysis, users can again choose from several plots including forest plots, and prior and posterior plots, as well as several tables including the model probabilities table and the study effect sizes table. Additionally, it is possible to conduct the analysis as a sequential analysis. Because these outputs are quite similar to the outputs described in the main text, here we will limit the explanation to the main results.

Table 11 contains the main results of the constrained random effects analysis, and it is similar to Table 2. The table shows the posterior estimates per model and Bayes factors to compare the different models of the analysis with each other. The estimates of *μ* are quite similar across models, and so are the estimates of *τ* from the ordered-effects and the random-effects models.

**Table 11:** Posterior estimates per model

				95% Credible Interval		
		Mean	SD	Lower	Upper	BF_1__0_
Fixed effects	*μ*	0.578	0.059	0.463	0.695	1.125e + 20
Ordered effects	*μ*	0.572	0.070	0.433	0.709	1.515e + 20^*a*^
	*τ*	0.097	0.065	0.019	0.258	1.347^*b*^
Random effects	*μ*	0.571	0.071	0.429	0.707	9.611e + 19
	*τ*	0.097	0.067	0.019	0.263	0.854^*b*^

*Note.* *μ* and *τ* are the group-level effect size and standard deviation, respectively

^a^ Bayes factor of the ordered effects ${\mathscr{H}}_{1}$ over the fixed effects ${\mathscr{H}}_{0}$. The Bayes factor for the ordered effects ${\mathscr{H}}_{1}$ versus the unconstrained (random) effects ${\mathscr{H}}_{1}$ model is 1.576

^b^ Bayes factor of the (unconstrained/constrained) random effects ${\mathscr{H}}_{1}$ over the fixed effects ${\mathscr{H}}_{1}$

The Bayes factors are shown in the last column. To properly interpret them it is necessary to again understand which models are considered in this analysis. The models considered here are the fixed-effects ${\mathscr{H}}_{0}$ and ${\mathscr{H}}_{1}$, the ordered-effects ${\mathscr{H}}_{1}$ where all study effects are constrained to be positive, and the random-effects ${\mathscr{H}}_{0}$ and ${\mathscr{H}}_{1}$. The Bayes factor in the first row of Table 11 is the evidence for the fixed-effects ${\mathscr{H}}_{1}$ over the fixed-effects ${\mathscr{H}}_{0}$; because we do not include the ordered-effects ${\mathscr{H}}_{0}$ for model comparison, the Bayes factor in the second row is the evidence for the ordered-effects ${\mathscr{H}}_{1}$ over the fixed-effects ${\mathscr{H}}_{0}$; the Bayes factor in the third row is the evidence for the ordered-effects ${\mathscr{H}}_{1}$ over the fixed-effects ${\mathscr{H}}_{1}$; the Bayes factor in the fourth row is the evidence for the random-effects ${\mathscr{H}}_{1}$ over the random-effects $\mathcal H_{0}$; and lastly, the Bayes factor in the fifth row is the evidence for the random-effects ${\mathscr{H}}_{1}$ over the fixed-effects $\mathcal H_{1}$. From these results we can see that the overall preferred model is the ordered-effects ${\mathscr{H}}_{1}$, but it is only preferred over the fixed-effects ${\mathscr{H}}_{1}$ by 1.347 to 1, and over the random-effects ${\mathscr{H}}_{1}$ by 1.576 to 1. We may therefore interpret the analysis as inconclusive. The data seemingly do not provide much evidence for the case that all studies have a positive effect, nor do they provide evidence for qualitative differences. The only thing we can conclude with certainty from this analysis is that there is a positive overall effect as evidenced by the large Bayes factors for the ${\mathscr{H}}_{1}$ over the ${\mathscr{H}}_{0}$ for all models.

## Footnotes

1 We acknowledge that the “fixed-effects” terminology is confusing, as the meta-analytic interpretation of the term “fixed-effects” differs from the definition of fixed effects in other modelling approaches in the statistics literature (Gelman & et al, 2005). Although we believe this meta-analytic model should rather be referred to as a “common-effect” model, in this paper we follow the standard terminology for meta-analysis.

2 Although the use of heterogeneity statistics is common practice, these tests often have low power and are therefore not recommended as a basis for deciding between the fixed-effects and the random-effects model (Stijnen, White, & Schmid, 2020). One advantage of Bayes factors is that they allow one to discriminate between ‘evidence of absence’ and ‘absence of evidence’. In the latter case (i.e., BF $\sim $ 1), we can say that the data are inconclusive. Note that such a conclusion cannot be drawn based on *p* values where the two cases are confounded. Furthermore, one of the major benefits of Bayesian model-averaging is that we may often not have enough data to conclude with certainty whether there is heterogeneity, but Bayesian model-averaging accounts for this uncertainty even if the data are inconclusive.

3 The model-averaged estimate of *τ* is not yet available in JASP.

4 A 95*%* highest density interval (HDI) is the smallest interval that contains 95*%* of the posterior distribution.

5 A detailed example of this analysis is demonstrated in Appendix B.

6 Similar as for the cumulative forest plot, a random-effects (or model-averaged) meta-analysis requires at least two studies. When the number of studies is one, the sequential plots indicate a Bayes factor of one.

## Code Availability

We analyzed the data with JASP (JASP Team, 2020). An annotated .jasp file, including plots, tables, data, and input options, is available at https://osf.io/84gbu/.
